# Evaluating the UK House of Commons Science and Technology Committee’s position on the implausible effectiveness of homeopathic treatments

**DOI:** 10.1007/s11017-017-9415-y

**Published:** 2017-07-04

**Authors:** Andrew Turner

**Affiliations:** Digital Ethics Lab, Oxford Internet Institute, 41 St Giles, Oxford, OX1 3JS UK

**Keywords:** Homeopathy, Mechanisms, Evidence-based medicine

## Abstract

In 2009, the UK House of Commons Science and Technology Committee (STC) conducted an ‘evidence check’ on homeopathy to evaluate evidence for its effectiveness. In common with the wider literature critical of homeopathy, the STC report seems to endorse many of the strong claims that are made about its implausibility. In contrast with the critical literature, however, the STC report explicitly does not place any weight on implausibility in its evaluation. I use the contrasting positions of the STC and the wider critical literature to examine the ‘implausibility arguments’ against homeopathy and the place of such arguments within evidence-based medicine (EBM). I argue that the STC report undervalues its strong claims about the mechanistic plausibility of homeopathy because it relies on a misunderstanding about the role of mechanistic evidence within EBM. This is not a conclusion for a revision of the role mechanistic evidence plays within EBM, however. It is a conclusion about the inconsistency of the STC report’s position towards implausibility arguments, given the evidential claims they endorse and the atypical situation that homeopathy presents. It provides a further example of the general point that mechanistic reasoning should not be seen as providing categorically lower quality evidence.

## Introduction

In the last 10 years, academic and public debate about homeopathy has renewed interest in whether it ‘really’ works, whether it is ethical to provide, and in the UK, what its proper place is within the National Health Service (NHS). In 2005, Shang et al. [1] published a meta-analysis of homeopathic treatments in the *Lancet*, concluding that there was no evidence that homeopathic treatments were better than placebo. This meta-analysis became a focal point for growing criticism of homeopathy and attracted many responses in the academic literature [2–14]. Since the publication by Shang et al., there have been popular science books [15–17], newspaper articles (see especially *the Guardian* 2007–2010 [18–23]), and public campaigns [24, 25], all aimed against the continued place of homeopathy in UK healthcare.

As a culmination of this critical movement against homeopathy, the UK House of Commons Science and Technology Committee (STC) conducted an ‘evidence check’ on homeopathy to investigate these questions, and published their findings in 2010 [26]. The STC have a remit to investigate the evidence that is used for Government policy and decision-making, and their report on homeopathy is an interesting document to consider for three reasons. First, it was produced at the peak of criticism of homeopathy in the UK. Although the report does not explain why the STC chose to examine Government policy around homeopathy in 2009–2010, the profile of wider criticism at the time is the likely reason. Second, the report provides an excellent synthesis of the wider criticism. In fact, the STC considered evidence submitted to them in writing and through two oral panel sessions that included some of the most prominent contributors to the public debate [26]. Third, the report does not simply repeat the wider criticism. In the STC report, as elsewhere, the counter-intuitive mechanism by which homeopathic treatment is supposed to work is criticised. However, the STC report draws a quite different conclusion from the apparent implausibility of homeopathy.

This difference in the way that the STC treats implausibility and the way the wider literature treats it is the focus of this article. Moreover, the focus is on the argument that the STC report contains rather than the circumstances that shaped the STC’s position. The question here is normative: how should the STC report treat implausibility?

First, I describe the difference between the way that implausibility arguments are used by the STC in their report and how they are used in the criticisms of homeopathy that are found in the wider literature. Second, I argue that the STC report endorses a puzzling position on the implausibility of homeopathy because it unfairly dismisses plausibility considerations in its evaluation, and because it relies on a misinterpretation of the role of mechanistic reasoning within evidence-based medicine (EBM). I argue that on the basis of its own assumptions (which may or may not be true), the STC report undervalues mechanistic evidence against the efficacy of the putatively pharmacological component of homeopathic treatment.

Whereas the wider critical literature contains a relatively stable and coherent set of statements about the mechanistic implausibility of homeopathic treatment that is largely carried over into the STC’s report, the STC report is far more cautious about the conclusions that can be derived from mechanistic reasoning. My argument is about the consistency of the position adopted in the STC report. It is independent of the actual state of the mechanistic evidence for or against the efficacy of homeopathic treatment. My conclusion is that the claims in the STC report are inconsistent because they are based on a misinterpretation of EBM: the misinterpretation is due to the assumption that mechanistic reasoning categorically provides low quality evidence. In fact, a better interpretation of the EBM view of mechanistic reasoning would actually allow the STC report to make their argument stronger. If the report’s claims about implausibility are true, then the report has undervalued the mechanistic evidence against homeopathy.

## Arguments about implausibility in the STC report and the wider critical literature

### What is implausible about homeopathy?

The STC report contains a series of arguments that are all negative towards homeopathy. The report presents arguments for the conclusion that homeopathic treatments do not work, as well as for the conclusion that homeopathic treatments should not be available. I focus only on one argument from the STC report. I am concerned with what I interpret as the claim that it is implausible that the ‘quasi-pharmacological component’ of homeopathic treatment could be effective. I use the term quasi-pharmacological component to highlight two important points (it is not the STC’s term).

First, the term highlights the counter-intuitive therapeutic theory behind the preparation of homeopathic medicines. Homeopathic medicines purport to contain an active component, albeit in an unconventional sense. This is because homeopathic medicines (that is, the pills themselves) have been exposed to a ‘potentised’ liquid produced through a process of progressive dilution and shaking (‘succusion’), which began with a substance that, at less dilute doses in healthy individuals, would cause the patient’s symptoms (this is the ‘like-cures-like’ or ‘similarity’ principle behind homeopathy). Homeopathic medicines are claimed to be more potent if they have been exposed to a potentised liquid that has gone through more of these dilution-shaking cycles (this is the ‘high-dilutions’ principle) [27–33]. Clearly, this contrasts with the more familiar way in which treatments are said to have active components, such as when they contain a substance in the way that a painkiller contains paracetamol. Conventionally, a drug treatment becomes more potent when there is more of the pharmacological component. Consequently, I refer to the active component of homeopathic treatment as its quasi-pharmacological component: homeopathic medicines have a sort-of pharmacological component, but one that is unusual because of the therapeutic theory behind it. Although the therapeutic theory behind homeopathy motivates critical arguments about the implausibility of its effectiveness, the use of the term ‘quasi-pharmacological’ is not intended to prejudice the discussion. I suggest that homeopathic medicines can be said to have a quasi-pharmacological component simply on account of the fact that the theory is unusual or unconventional, without saying anything further about plausibility.

Second, referring to the quasi-pharmacological component of homeopathic treatment highlights the fact that it is only one of many other components. Treatments are rightly viewed as a package of components made up of, for example, bulking agents, pill casings, drug contents, delivery mechanisms, etc., each of which may or may not be individually effective for the target condition [34–36] (and which may also interact with each other). Even if the quasi-pharmacological component of homeopathic treatment is not effective, this does not necessarily mean that the treatment as a whole is ineffective. In fact, the STC report acknowledges that idea by claiming that homeopathic treatments are effective through placebo effects [26]. That is one way to express the idea that some components of a treatment may be efficacious (the components that give rise to placebo effects), while others are not.

In these terms, then, this article is concerned with the argument found in both the wider critical literature and in the STC report, namely, that the purported effectiveness of the quasi-pharmacological component of homeopathic treatment is implausible. In general, the implausibility argument against homeopathy denies that there is a mechanism by which the quasi-pharmacological component of homeopathic treatments could possibly work, though this is deployed differently in the STC report and the wider literature.

In the next section, I describe the arguments about implausibility as they are found in the wider literature that criticises homeopathy, noting the strength of the conclusion that it most often puts forward. The argument might be better called an ‘impossibility’ argument, which is an important indicator of the rhetorical role that the strong conclusion in the literature also plays. I then describe the discussion of implausibility as it appears in the STC report. The key point is that, despite agreement between the STC and the wider critical literature on the evidence for implausibility, the STC take a substantially weaker position on the relevance of this evidence.

### Implausibility in the critical literature about homeopathy

Although many of the criticisms of homeopathy received their fullest expression in the STC report, they have been in circulation and developed in the academic literature and public media prior to it. Indeed, many have been present throughout the 200 years of criticism that homeopathy has received since it was first conceived. In fact, the implausibility of the effectiveness of the quasi-pharmacological component of homeopathic treatment was noted in the 19th century. For example, John Forbes (who is more charitable about assessing the claims of homeopaths on their own terms than many others) describes the problems with the idea that homeopathic treatments could be effective as follows:

to admit the potency of homeopathic medicaments is not so easy. Indeed, it is so difficult, that all the arguments that have hitherto been adduced in support of the affirmative of the proposition [that homeopathy works], are incapable of making any impression on ordinary minds, while the glaring improbability of the fact lies open before them.… [T]he reasons against the doctrine are so manifold and obvious that it is almost unnecessary to state them…. [The mechanism] seems so gratuitous an outrage to human reason that the mind instinctively recoils from the proposition. [37, pp. 16–17]

Today, echoing Forbes, similar critical emphasis is placed on the idea that a substance can get more potent as it is diluted. Timothy Caulfield and Suzanne Debow conducted a systematic review of how homeopathy was represented in conventional and alternative medicine journals. They found that within conventional medical journals, nine out of ten review articles ‘begin with a statement that questions the scientific plausibility of homeopathy’ [38]. Statements of implausibility are common in many other articles about homeopathy, not just systematic reviews. Consider some of the most prominent examples:

It is in particular the use of highly diluted material that overtly flies in the face of science. [39]

There is no notion in chemistry or biology that would explain such an effect…. So, clearly, that is not how homeopathic remedies could work. They do not work by retaining any active ingredient. [40]

[Homeopathy is] not only out of line with scientific facts but also directly opposed to them. If homeopathy is correct, much of physics, chemistry, and pharmacology must be incorrect.… We think that a belief in homeopathy exceeds the tolerance of an open mind. We should start from the premise that homeopathy cannot work. [41]

It is a cure that does not make sense in the light of science.… [H]omeopathy cannot possibly work because it has no scientifically plausible grounds. [42]

Those who claim that homeopathy is effective have enormous unexplained mysteries, and answering those mysteries would appear to require massive revision of standard chemistry and physiology…. [T]he balance is heavily against homeopathy. [43]

We understand that it cannot work through any mechanism that is in accordance with the known laws of nature. [44]

These are strong claims. The strongest suggest that homeopathy ‘cannot’ work. Other statements make a slightly weaker claim by allowing the *possibility* that the quasi-pharmacological component of homeopathy is effective, but balance this concession against the substantial revisions in our understanding of the sciences that they claim would be necessary as a result.

The crucial move in this contemporary critical literature is to take the implausibility of a mechanism by which the quasi-pharmacological component of homeopathic treatments could be effective as evidence that it is not effective. This idea is at the heart of what I call an ‘implausibility argument against homeopathy’, which goes as follows:

1. It is very implausible that there could be a mechanism by which the quasi-pharmacological component of homeopathic treatment is effective.
2. If it is very implausible that there could be a mechanism for how some component of a treatment is effective, then it is very unlikely that component is effective.
  Therefore,
3. It is very unlikely that the quasi-pharmacological component of homeopathic treatments is effective.

The critical literature contains many statements like those quoted above that demonstrate a commitment to the claims made in premise (1) and the conclusion. It is harder to find a clear statement of premise (2). The move from (1) to (3) is not spelt out in the literature.

There is some ambiguity in the statements quoted above around whether they are statements of implausibility or would be better characterised as statements of impossibility. Those claiming that homeopathy ‘cannot’ work are easily read as statements of impossibility. If ‘very implausible’ is changed to ‘impossible’ in the argument above, then it remains valid. However, that also significantly raises the evidential burden. The argument becomes invalid if one tries to use (1) and (2) as they stand to draw the conclusion that it is impossible that the quasi-pharmacological component of homeopathic treatment is effective. Equally, premise (2) becomes false if it is modified to allow one to move from a very implausible mechanism to actual ineffectiveness, rather than to very implausible effectiveness.

Reconstructing the argument also requires some sensitivity to the status of the texts these quotes come from. The strong statements of impossibility are typically found in editorials and commentary articles, so it is important to acknowledge the rhetorical force that a claim of this strength possesses, and therefore, to recognise the role that this kind of discourse plays in attempts to close the debate by exaggerating the evidence. I suggest that the most charitable construction of the argument is to put it in terms of implausibility rather than impossibility. Whether the argument is spelt out in terms of impossibility or implausibility, it is important that we should not be able to infer that the quasi-pharmacological component of homeopathy *cannot* be effective simply because we believe that it *should* not be effective. We are not infallible and the implausibility argument does not claim that we are.

There are two further points to note about this implausibility argument. First, the argument is based on inferences from theory rather than from comparisons between groups of individuals; hence, the suggestions that homeopathy is in conflict with ‘known laws of nature’, ‘standard chemistry and physiology’, or ‘much of physics, chemistry and pharmacology’ (indeed all the basic sciences—physics, chemistry, biology—are referenced in one or more of the quotes above). The inference is from basic theoretical knowledge that it is implausible that there could be a mechanism by which the quasi-pharmacological component of homeopathy works to the conclusion that it likely does not work.

Second, it is easy to misunderstand the inference. The arguments made in the wider critical literature about homeopathy do not conclude that the quasi-pharmacological component *may* be effective because one is in a position of mechanistic ignorance. That is to say, the conclusion is not that one does not know or cannot be sure that the quasi-pharmacological component is effective because we lack a full mechanistic understanding of it. It is an argument for the stronger conclusion that mechanistic implausibility provides good evidence that the quasi-pharmacological component of the treatment is very unlikely to be effective. The claim is that mechanistic implausibility constitutes evidence for the likely absence of effectiveness.

### Implausibility in the STC report

The STC endorse premise (1). In common with the wider critical literature, the STC report endorses similarly strong claims about the implausibility of a mechanism by which the quasi-pharmacological component of homeopathic treatment could be effective. The key statements in the report occur in paragraphs 48–62, in a substantial section titled ‘Scientific Plausibility for a Mode of Action’. In this section of the report, two of the theoretical principles behind homeopathic treatment that inform the production of homeopathic medicine are scrutinised. The first of these is the similarity principle, which is the idea that ‘like-cures-like’, noted above. The second is the notion of potentisation, also noted above, and in particular, the high level of dilution of the potentised liquids that are used to produce homeopathic medicines.

The similarity principle and the use of high dilutions are both regarded as scientifically implausible. For example, the similarity principle is claimed to be ‘theoretically weak’: ‘It fails to provide a credible physiological mode of action for homeopathic products’ [26, para. 54]. Similarly, the notion of high dilutions is claimed to give rise to ‘enormous difficulties’: ‘Even if water could retain a memory of previously dissolved substances, we know of no explanation for why the sugar-based homeopathic pills routinely dispensed would retain such a memory…. We consider the notion that ultra-dilutions can maintain an imprint of substances previously dissolved in them to be scientifically implausible’ [26, paras. 60–61].

To support these claims, the report cites evidence submitted to the STC (either in writing or during their panel sessions). For example, they quote favourably the view of David Colqhoun, a professor of pharmacology and prominent critic of homeopathy: ‘If homeopathy worked, the whole of chemistry and physics would have to be overturned’ [26]. The report also quotes the view of Jayne Lawrence, Chief Scientific Advisor to what was at the time the Royal Pharmaceutical Society of Great Britain: ‘I think it probably would be revolutionary if homeopathy was proved to be right, because it does go against a lot of fundamental understanding of science as it stands at the moment’ [26, para. 59]. Again, in common with the wider critical literature, these claims about the implausibility of the mechanism by which the quasi-pharmacological component of homeopathic medicines could be effective rely on the idea that such a mechanism would be ‘far removed from current scientific understanding’ [26, para. 59].

As noted above, strong statements of implausibility play an important rhetorical role by allowing critics of homeopathy to close down the debate about its effectiveness as settled. This strategy is also apparent in the STC report when it questions whether it is appropriate to invest in further research ‘exploring theories that are not scientifically plausible’. Moreover the STC heard evidence from the Government Chief Scientific Adviser, who claimed that there is no evidence base for homeopathy. This led to the STC further recommending that the UK Government and Department of Health jointly discuss whether there is ‘any merit in research funding being directed towards the claimed modes of action of homeopathy’ [26, para. 64]. By questioning the value of funding further research into homeopathy, the STC report, like the wider critical literature, uses evidence of implausibility to suggest that the question of whether there is a plausible mechanism is closed.

I claim, therefore, that the STC report holds the same position as the wider critical literature in relation to the key evidential premise (1) in the implausibility argument above. The report endorses the view that it is very implausible that there could be a mechanism by which the quasi-pharmacological component is effective.

Unlike the wider critical literature, however, the STC reject the use of premise (1) to make an argument against the effectiveness of the quasi-pharmacological component of homeopathy. The STC report does not appear to endorse the conclusion of the implausibility argument. Instead, they hold the view that the implausibility of a mechanism by which the quasi-pharmacological component could be effective counts for very little in the assessment of whether it is effective. To illustrate, the STC goes on to de-emphasise the role that plausibility considerations play in their assessment of the evidence for the effectiveness of homeopathy: ‘While we comment on explanations for how homeopathy works, it is not a key part of our Evidence Check’ [26, para. 18]. The STC defend this position by stating that ‘historically, some medical interventions were demonstrably effective before anyone understood their modes of action’ [26, para. 18], and that the ‘lack of scientific plausibility is disappointing, but does not necessarily mean that a treatment does not work’ [26, para. 65]. Here, the STC report’s reason for not making plausibility a key part of its evidence check is that one does not need to understand how a treatment works in order to know that it is effective, and the two quotes above express this as a historical and conceptual truth. Since such evidence is unnecessary, it is not considered by the STC.

In sum, the STC agree with the wider critical literature that the effectiveness of the quasi-pharmacological component of homeopathy is very implausible. The key difference between the STC report and wider critical literature is that the STC report does not value and subsequently use this evidence in their assessment of the effectiveness of homeopathy. Given the rhetorical force of the implausibility argument to close down the debate, this deliberate discounting of such evidence is particularly noteworthy. The STC defend their rejection of plausibility considerations in their evaluation on the basis that evidence of plausibility is not necessary for the effectiveness of the quasi-pharmacological component of homeopathic treatment.

## Evaluating the STC position on implausibility

### Absence of evidence and evidence of absence

The STC report is correct that lack of evidence of plausibility should not be taken to rule out the effectiveness of the quasi-pharmacological component. However, stating that fact, as they do in the quotations above, is not a denial of premise (2); moreover, it does not engage with the kind of claim being made in premise (1). Although true, it does not provide a good reason to reject the implausibility argument. The difference between the implausibility argument and the STC’s rejection of plausibility considerations is similar to the difference between ‘evidence of absence’ and ‘absence of evidence’.

To reiterate the premises of the implausibility argument, premise (1) is that it is very implausible that *there could be a mechanism* by which the quasi-pharmacological component of homeopathic treatment is effective. Premise (2) makes a link between the very implausible possibility of a mechanism and the likely ineffectiveness of that component of a treatment. The implausibility of there being any mechanism by which the quasi-pharmacological component could be effective is taken to provide good evidence that the component likely is not effective. The implausibility argument therefore aims to establish evidence of absence.

This is a separate issue from whether there might be a mechanism that we do not know or understand, and it is unrelated to the fact that one does not need a treatment to seem plausible in order to know that it works. The STC report is right to state that absence of evidence of plausibility is poor grounds to deny the effectiveness of the quasi-pharmacological component of homeopathy. The STC are wrong, however, to say that this therefore provides a good reason to dismiss the implausibility argument. The implausibility argument is a much stronger argument based on the notion that there is unlikely to be any possible mechanism by which the quasi-pharmacological component could be effective.

The first problem facing the STC’s position, therefore, is that the reasons they provide for rejecting the relevance of plausibility considerations in general are not good reasons for rejecting the implausibility argument. Additionally, there is also a second, more interesting, problem for the STC position. Not only has the STC report misunderstood the implausibility argument, but it has also undervalued the role of mechanistic reasoning in the assessment of homeopathy. I argue that the STC have missed the opportunity to make the argument in their report *stronger*.

### The role of mechanistic reasoning

The STC do not value implausibility as evidence. This can be further seen in the way the report deploys a distinction between whether and how a treatment works. The report states: ‘It is more important to know *whether* a treatment works—its efficacy—than *how* it works’ [26, para. 18]. Here, a distinction is made between the mechanistic question of ‘how’ a treatment works, and the clinical question of ‘whether’ a treatment works. By making such a distinction, the STC report establishes the lower importance of mechanistic reasoning.

Furthermore, elsewhere in the STC report, there are clear expectations for what kind of evidence is most important and should count in the assessment of the effectiveness of the quasi-pharmacological component of homeopathy. Namely, the best evidence comes from the pooled results of randomised trials using a placebo control. As the report states, ‘In clinical research, it is widely accepted that RCTs are the best way to evaluate the efficacy of different treatments and distinguish them from placebos…. We consider that conclusions about the evidence on the efficacy of homeopathy should be derived from well designed and rigorous randomised controlled trials (RCTs)’ [26, para. 20]. The report elsewhere states: ‘What is important is how a treatment performs when tested fairly against a placebo treatment or other treatments. We consider that the best evidence is provided by randomised controlled trials, meta-analyses and systematic reviews of RCTs [26, para. 65].

This position is, of course, exactly what one would expect from a commitment to EBM. EBM provides the framework for the report’s statements about the ‘wide acceptance’ of randomised trials as providing good evidence of treatment effects. Indeed, a placebo-controlled randomised trial is perfectly equipped to assess the effectiveness of the quasi-pharmacological component of homeopathic treatment. Although the STC report does not cite EBM textbooks or any of the current evidence rating systems (for example, the OCEBM levels [45] or GRADE [46]), the emphasis on results from single or pooled randomised trials and explicit rejection of mechanistic questions in favour of clinical questions is clearly very close to typical EBM accounts about what counts as good evidence for treatment benefit.

Given that the STC report rejects plausibility considerations, and given that I have claimed that the reasons the STC provide do not warrant this rejection, the question is whether there are further resources within the EBM paradigm that permit the STC to dismiss the implausibility argument. I argue no; in fact, I argue that homeopathy is an unusual case because it is an example of a case in which the EBM view should be to embrace the implausibility argument.

#### Mechanistic reasoning and the implausibility argument

The medical literature on EBM has tended to give only a minor role to mechanistic reasoning about treatment effects, but it has allowed that such reasoning may be useful in other ways. For example, mechanistic reasoning is operative when generalising results from study populations to some target population [47]. However, the concession that mechanistic reasoning may be useful for generalising results has recently been challenged [48]. Jeremy Howick provides a refinement of the EBM account of mechanistic reasoning and argues that ‘the EBM position [where relatively little weight is given to mechanistic reasoning] is acceptable on the whole, [but] mechanistic reasoning can provide strong evidence in certain well-defined cases’ [49, p. 120]. Contrary to this, other philosophers considering mechanisms have argued that mechanistic reasoning should be given a more substantial evidential role within EBM. Perhaps the most discussed claim is one made by Federica Russo and Jon Williamson: ‘To establish causal claims, scientists need the mutual support of mechanisms and dependencies’ [50].

How do these debates bear on the implausibility argument and my interpretation of the STC position? Giving a greater role to mechanistic reasoning could support the kinds of claim made in the critical literature about homeopathy. If mechanistic reasoning is given a greater role, then that provides grounds to consider implausibility in an evaluation of the effectiveness of the quasi-pharmacological component of homeopathy. Furthermore, if the traditionally dominant view in EBM is that mechanistic reasoning counts very little, this would seem to explain the position taken in the STC report, because by sticking to this interpretation, they are not able to utilise that evidence.

Contrary to this approach, I focus in what follows on Howick’s view, which is more cautious about the role given to mechanistic reasoning. The reason for focusing on Howick’s view is to examine the implausibility argument and the STC report’s position without requiring substantive revisions to the EBM position on mechanistic reasoning. While giving mechanistic reasoning a much greater evidential role in EBM is, of course, one position from which to examine the debate about homeopathy, I suggest that it is fairer to consider a less radical account of what the proper role of mechanisms are in EBM. Howick’s view is particularly helpful in this regard because he provides criteria for when mechanistic reasoning should be considered good evidence.

First then, consider Howick’s definition of ‘mechanistic reasoning’: ‘[mechanistic reasoning] involves an inference from mechanisms to claims that an intervention produces a patient-relevant outcome. Such reasoning will involve an inferential claim linking the intervention (such as antiarrhythmic drugs) with a clinical outcome (such as mortality)’ [49]. This account of mechanistic reasoning is quite different from the implausibility argument. The way that mechanisms and mechanistic reasoning are discussed in the philosophy literature focuses on the presence of a mechanism underwriting some purported effect. For example, the quote above is illustrated with an example of an inference from knowledge of a drug treatment’s mechanisms to an effect on mortality. Similarly, in other works on mechanisms, the focus has been on the presence of mechanisms and establishing causal claims.

The implausibility argument has a different form, however. Reasoning about implausibility involves an inference from the absence of a mechanism to the absence of an effect. The inference is not from the existence of a known mechanism but from the implausibility that there could be a mechanism, and not to some effect but to the implausibility of there being any effect. The implausibility argument is also more specific, in the sense that it is not the effect of a treatment that is in question but the effect due to one particular feature of the treatment: the quasi-pharmacological component. The implausibility argument leaves open the possibility that there are other mechanisms by which homeopathic treatment is effective (one obvious example being expectation effects).

This account also helps clarify a crucial point made above, namely, that the implausibility argument is not an argument from ignorance: the inference is based on the claimed absence, not our ignorance, of a mechanism. Indeed, as shown above, the STC report and other critics of homeopathy claim that there is a significant level of empirical support for the implausibility of there being a mechanism by which the quasi-pharmacological component of homeopathic treatment could be effective.

The implausibility argument fits the broad pattern of mechanistic reasoning in so far as it is an inference from a claim about mechanisms to a claim about effects. But it is also clearly different from other examples because it is about the lack of any mechanism and the lack of an effect. Howick suggests that his criteria for judging the quality of mechanistic reasoning should apply to both acceptance and rejection of an effect [49]. Here though, there is some ambiguity about whether this might mean that knowledge of the presence of a specific mechanism can be good evidence that a treatment cannot have a particular effect, or whether it might mean that knowledge that there cannot be a mechanism for a particular effect is good evidence that there is no such effect. It is the latter that is closer to what the implausibility argument claims. Although Howick does not discuss in detail any examples similar to the implausibility argument, he does offer the brief suggestion that the reasoning ‘used to question Leibovici’s hypothesis was of high quality’ [49]. This hypothesis is worth considering in more detail, because it has much in common with the argument made about homeopathy.

Leonard Leibovici reported results from a randomised trial investigating the effect of remote, retroactive, intercessory prayer on patients who suffered from bloodstream infections [51]. The article was humorous and published as part of the *British Medical Journal*’s Christmas edition. Like the best articles in the Christmas editions, it makes a serious point with an absurd example. Leibovici examined the records of 3393 patients who were treated for bloodstream infections between 1990 and 1996 and randomised into two groups *in the year 2000*, one of which was chosen to be prayed for. Leibovici found that while intercessory prayer had no significant effect on mortality, the intervention group showed a small effect on secondary outcome measures: they had a statistically significant shorter duration of fever and a shorter stay in hospital (these two outcomes are not entirely independent).

Putting aside independent methodological reasons to reject the results of this study [49], an obvious concern is mechanistic implausibility. On the basis of very general and fundamental background knowledge about the world, we know that one cannot, in the present, cause events that happened in the past, and therefore, we know that there is no mechanism by which remote intercessory prayer could cause therapeutic effects. (Indeed, the fact that the intervention cannot possibly be the explanation of the result is what makes Leibovici’s article instructive.)

This result has strong parallels with what critics claim about the quasi-pharmacological component of homeopathy. Analogously to ruling out Leibovici’s hypothesis on the basis of general knowledge about causality, we also have general and fundamental background knowledge of physics, chemistry, and biology—so the critics argue—that rules out the possibility of there being a mechanism by which the quasi-pharmacological component of homeopathy could be effective. If we take remote retroactive prayer as the component of interest, we can run through the implausibility argument as set out in the above subsection, ‘Implausibility in the Critical Literature about Homeopathy’, unchanged. Leibovici’s hypothesis and the effectiveness of the quasi-pharmacological component of homeopathy can both be subjects of an implausibility argument.

Howick suggests that the mechanistic reasoning used to reject Leibovici’s hypothesis is likely to be high-quality. I claim that the parallels between Leibovici’s hypothesis and the implausibility of homeopathy warrant considering whether the implausibility argument against homeopathy might also count as high-quality reasoning. This is despite the fact, as outlined above, that the mechanistic reasoning involved does not proceed in the typical way from presence of mechanism to presence of effect.

#### Does the implausibility argument against homeopathy constitute high-quality mechanistic reasoning?

Howick argues that mechanistic reasoning ought to fulfil two criteria if it is to be counted as good evidence for a treatment effect. (Actually Howick is stricter in requiring not just any effect but an effect that is patient-relevant.)

- The knowledge of mechanisms upon which the mechanistic reasoning is based is *not incomplete*, i.e., there are no obvious gaps in our knowledge of the inferential chain linking the intervention and the patient relevant outcome.
- The probabilistic and complex nature of mechanisms are explicitly taken into account when inferring from mechanisms to any claims that a particular intervention has a patient-relevant benefit. [49]

Neither of these criteria fit the implausibility argument perfectly. In both cases, it is assumed that mechanistic reasoning is being used to link an intervention to an effect. However, these criteria can be adapted for the absence of a mechanism. The first criterion, ‘not incompleteness’ bears on the implausibility argument, I suggest, by requiring evidence for premise (1) to be genuine evidence of absence and not absence of evidence. The claim that it is very implausible that there could be a mechanism should not rely on lack of knowledge about possible mechanisms, but rather, should rely on ‘not incomplete’ knowledge of why such a mechanism is very implausible. Leibovici’s study provides a good example of this: it is very implausible that remote retroactive prayer could be effective because *any* mechanism would have to contradict, or be a special exception to, the general and fundamental idea that causes precede their effects. That one cannot now cause events in the past is an example of the kind of ‘not incomplete’ knowledge that is required by Howick’s criterion.

The second criterion, that complexity should be accounted for, is less straightforward to adapt. The spirit of this criterion comes from the observation by Howick that mechanisms are rarely, if ever, simple systems and many difficult-to-predict factors potentially affect whether a mechanism goes through. Consequently, I suggest that the evidence on which we claim that there cannot be a mechanism must be sensitive to the complexity that might exist between treatment and effect. To illustrate how this might affect plausibility arguments, consider that one might defend Leibovici’s result against apparent implausibility by noting that the supernatural properties of prayer exempt it from considerations based on cause and effect in the natural world. Proponents of homeopathy often make a similar kind of manoeuvre by invoking the complex physical–chemical properties of water. They often respond to implausibility claims with counter-arguments based on the macro-structures, fractals, or quantum properties of water [28, 52, 53].^1^ The important question that Howick’s second criterion prompts in the case of the implausibility argument is whether there is reason to think that homeopathy provides a special exception to general knowledge about dilutions and potency and so on. Is there some degree of complexity in the link between the treatment and purported effect that could undermine the idea that the possibility of a mechanism really is very implausible?

Given what both the STC report and the wider critical literature state about the implausibility of a mechanism by which the quasi-pharmacological component of homeopathy could be effective, does this qualify as high-quality evidence? I claim it does. To repeat, the STC claim that a mechanism would be ‘far removed from current scientific understanding’, and that homeopathic principles are ‘theoretically weak’ and present ‘enormous difficulties’. The claim in both the wider literature and the STC report is that there is good evidence that there cannot be a mechanism, not that there is no evidence for what the mechanism could be. If those claims are true (as the STC claim they are), then this must count as high-quality mechanistic reasoning. The claims that a mechanism for the effectiveness of the quasi-pharmacological component would contradict fundamental principles of physics, chemistry, and biology constitutes the kind of ‘not incomplete’ knowledge that fulfils Howick’s first criterion. The knowledge we have that (critics claim) allows one to rule out a mechanism for homeopathy is among our most fundamental background knowledge.

Furthermore, fulfilling Howick’s second criterion, this knowledge is sufficiently general, so critics claim, that we can confidently assert that it is very implausible that there could be a mechanism without worrying that there may be some unconsidered or unknown mechanisms by which the quasi-pharmacological component could, in fact, be effective. Critics claim there is nothing complex about homeopathy that makes it a special exception. This is what motivates those very strong rhetorical claims that homeopathy ‘cannot’ work: the claim is that our evidential position is sufficiently strong to make the effectiveness of homeopathy a closed question.

It is important to note that I am not endorsing this as the correct evidential position to take. I claim that the implausibility argument constitutes high quality mechanistic reasoning *if* one adopts the evidential position towards the implausibility of homeopathy that the STC adopt in their report, and which is also adopted in the wider critical literature. This is a conditional claim. I am not arguing that mechanistic reasoning about homeopathy should be taken as strong evidence. I am instead arguing that *if one believes what the STC endorse in their report*, then one should take mechanistic reasoning about homeopathy as strong evidence. Consequently, I claim that the STC have made a mistake by not doing this.

## Conclusion

The STC endorse the same evidential position as the wider critical literature about homeopathy. Both claim that it is very implausible that there could be a mechanism by which the quasi-pharmacological component of homeopathic treatment is effective. Unlike the wider critical literature, the STC do not build this into an ‘implausibility argument’ against homeopathy. They reject using plausibility considerations. I have argued that they do not give good reasons for their rejection of plausibility considerations.

Furthermore, I have argued that the rejection of plausibility considerations does not follow from Howick’s refinement of the role that mechanisms should play in EBM. The STC report adopts the typical EBM view that mechanistic reasoning counts for very little. If homeopathic treatments were like other conventional drug treatments, then this would be the acceptable position to hold. The case of homeopathy is different however because of what the STC report claims about its implausibility. I have argued that the report’s statements about implausibility fulfil Howick’s criteria for being high-quality mechanistic reasoning (assuming that what the STC claim is true). As a result, evidence of implausibility should count in evaluations of whether the quasi-pharmacological component is effective, contrary to the approach taken in the STC report. On the basis of their own assessment of the evidence, the STC should have been able to make the implausibility argument. In doing so, they could have strengthened their case against homeopathy.

There are two important points to note about this conclusion. Firstly, as I have emphasised, this conclusion is conditional. The STC can make a stronger argument only if their evidential claims are warranted. In fact, proponents of homeopathy may be in the better epistemic position here, since they only need to prevent the efficacy of the quasi-pharmacological component of homeopathy being ruled out to put the mechanistic debate ‘on the table’. That is to say, unless the strong evidential premise in the implausibility argument can be justified, it is not a sound argument. I have not discussed the to and fro between opponents and proponents of homeopathy, however, a key aspect of the controversy is the use of statements about plausibility to frame certain questions as open or closed, and to shift the burden of evidence in precisely this way. Again, coming back to the interaction between philosophical and sociological issues, an interesting epistemological question is whether a dialectical standard of evidence is appropriate in the debate over the assessment of homeopathy [54].

Secondly, this conclusion is fundamentally about the consistency of the position adopted in the STC report. By adhering to a dismissive view of mechanistic reasoning (which is typically the correct view), the STC report has failed to make the most of its own strong evidential claims. Consequently, the STC report’s position on the implausible effectiveness of homeopathic treatments provides a further example of the more general point that mechanistic reasoning should not be seen as providing categorically lower quality evidence. The implication of this is that any evaluation of the evidence for the efficacy of the quasi-pharmacological component of homeopathic treatment ought to take into account the mechanistic evidence for and against its purported efficacy. For homeopathy, and unlike most conventional treatments, the purported mechanisms and principles behind the treatment are directly relevant to the debate, if, as critics claim, they can be ruled out as highly implausible.

## Footnotes

1 Of course, this evidence itself is a form of mechanistic reasoning that one could evaluate using Howick's criteria. The validity of this to and fro between proponents and opponents of homeopathy is not the concern here, however.
